# Variability and Reliability of Paired-Pulse Depression and Cortical Oscillation Induced by Median Nerve Stimulation

**DOI:** 10.1007/s10548-018-0648-5

**Published:** 2018-05-08

**Authors:** Hideaki Onishi, Naofumi Otsuru, Sho Kojima, Shota Miyaguchi, Kei Saito, Yasuto Inukai, Koya Yamashiro, Daisuke Sato, Hiroyuki Tamaki, Hiroshi Shirozu, Shigeki Kameyama

**Affiliations:** 1 Institute for Human Movement and Medical Sciences, Niigata University of Health and Welfare, 1398 Shimami-cho, Kita-ku, Niigata, 950-3198 Japan; 2 Department of Functional Neurosurgery, Nishi-Niigata Chuo National Hospital, 1-14-1 Masago, Nishi-ku, Niigata, 950-2085 Japan

**Keywords:** Magnetoencephalography, SEF, Sensory gating, Event-related desynchronization, Event-related synchronization

## Abstract

Paired-pulse depression (PPD) has been widely used to investigate the functional profiles of somatosensory cortical inhibition. However, PPD induced by somatosensory stimulation is variable, and the reasons for between- and within-subject PPD variability remains unclear. Therefore, the purpose of this study was to clarify the factors influencing PPD variability induced by somatosensory stimulation. The study participants were 19 healthy volunteers. First, we investigated the relationship between the PPD ratio of each component (N20m, P35m, and P60m) of the somatosensory magnetic field, and the alpha, beta, and gamma band changes in power [event-related desynchronization (ERD) and event-related synchronization (ERS)] induced by median nerve stimulation. Second, because brain-derived neurotrophic factor (BDNF) gene polymorphisms reportedly influence the PPD ratio, we assessed whether BDNF genotype influences PPD ratio variability. Finally, we evaluated the test–retest reliability of PPD and the alpha, beta, and gamma ERD/ERS induced by somatosensory stimulation. Significant positive correlations were observed between the P60m_PPD ratio and beta power change, and the P60m_PPD ratio was significantly smaller for the beta ERD group than for the beta ERS group. P35m_PPD was found to be robust and highly reproducible; however, P60m_PPD reproducibility was poor. In addition, the ICC values for alpha, beta, and gamma ERD/ERS were 0.680, 0.760, and 0.552 respectively. These results suggest that the variability of PPD for the P60m deflection may be influenced by the ERD/ERS magnitude, which is induced by median nerve stimulation.

**Electronic supplementary material:**

The online version of this article (10.1007/s10548-018-0648-5) contains supplementary material, which is available to authorized users.

## Introduction

The paired-pulse stimulation (PPS) paradigm has been widely used to investigate the functional profiles of somatosensory cortical inhibition, known as paired-pulse depression (PPD), pre-pulse inhibition, or sensory gating, using auditory or somatosensory stimulation (Braff et al. 2001; Cheng et al. 2016, 2017; Huttunen et al. 2008; Swerdlow et al. 2005). PPD is induced by stimulating with two pulses separated by a 100–2000 ms inter-pulse interval (ISI) (Cheng et al. 2016, 2017; Onishi et al. 2016; Xu et al. 2009). It is evaluated as the amplitude ratio of the second stimulus (test stimulus, TS) response to the first stimulus (conditioning stimulus, CS) response. Intracellular recordings have shown that γ-aminobutyric acid (GABA) is involved in PPD (Davies and Collingridge 1993; Davies et al. 1990; Deisz and Prince 1989; Xu et al. 2009). In human studies, drug-related PPD changes have shown that GABA is related to PPD (Huttunen et al. 2008; Stude et al. 2016). Moreover, PPD is reduced in elderly people (Goto et al. 2015; Lenz et al. 2012), and GABA concentrations decrease with age (Grachev et al. 2001). In addition, PPD induced by somatosensory stimulation is related to the degradation of tactile perception (Lenz et al. 2012; Rocchi et al. 2017). However, in human studies, PPD induced by somatosensory stimulation is variable, and is sometimes deficient even in healthy young subjects (Lenz et al. 2012; Onishi et al. 2016). In addition, the reason for between- and within-subject variability of PPD induced by somatosensory stimulation remains unclear. Thus, the reliability of PPD measures must be demonstrated before implementing the use of inhibitory functions such as clinical biomarkers (e.g., degradation of tactile perception or GABA concentration in the primary somatosensory cortex). If PPD measurements vary between sessions, the statistical power of these measurements is decreased, which limits the robustness of conclusions made regarding the effects of the studied drug, treatment, or disease.

Numerous studies have reported the relationship between PPD, cortical oscillatory activity, and GABA concentration. For example, PPD is well known to be disrupted in schizophrenia (Swerdlow et al. 2008), and ongoing gamma band oscillations are higher in schizophrenics (Spencer 2011; Tatard-Leitman et al. 2015). NMDA receptor antagonists, such as Ketamin and MK-801, are known to increase ongoing gamma frequency power and reduce PPD (Ehrlichman et al. 2009; Jones et al. 2014; Kulikova et al. 2012). Beta band oscillations are also related to PPD and GABA concentration. Indeed, among GABAa agonists, diazepam and benzodiazepine increase beta frequency power (Hall et al. 2010; Jensen et al. 2005) and Lorazepam reduces PPD (Huttunen et al. 2008; Stude et al. 2016). GABA concentrations are positively correlated with beta frequency power (Baumgarten et al. 2016). Moreover, post-movement or post-stimulus beta rebound (event-related synchronization, ERS) is positively correlated with GABA concentration (Gaetz et al. 2011), and beta ERS is associated with PPD (Cheng et al. 2017). On the other hand, it has been reported that beta band event-related desynchronization (ERD) is facilitated by the GABA agonist diazepam, whereas beta ERS is not affected by the drug (Hall et al. 2011). Moreover, tiagabine, which blocks GABA transporters and increases endogenous GABA activity, enhances beta ERD and reduces beta ERS (Muthukumaraswamy et al. 2013). In addition to gamma and beta oscillation, it has also been reported that alpha ERD is associated with age-related PPD reduction (Cheng et al. 2015). These reports suggest that PPD variability may be closely related to the magnitude of alpha, beta, or gamma ERD/ERS induced by somatosensory stimulation. In this study, we investigated the relationship between the PPD ratio and the alpha, beta, and gamma ERD/ERS magnitude induced by somatosensory stimulation in order to clarify the between-subject PPD variability.

In human studies, a scalp level signal for PPD has been detected using magnetoencephalography (MEG) or electroencephalography (Cheng et al. 2016; Huttunen et al. 2008; Jones et al. 2014; Lenz et al. 2012; Nakagawa et al. 2014; Onishi et al. 2016). The somatosensory evoked magnetic fields (SEF) and somatosensory evoked potentials elicited by stimulation of the peripheral nerves, such as the median nerve, return to baseline approximately 300 ms after stimulation. However, peripheral nerve stimulation leads to decreases in alpha and beta band power (ERD), which is followed by increases in power above the baseline level (ERS). Peak alpha and beta ERD is observed approximately 300 ms after median nerve stimulation at the wrist, and ERS occurs approximately 400 ms after stimulation and lasts for about 1000 ms (Dockstader et al. 2008; Salenius et al. 1997; Schnitzler et al. 1997). Thus, under a PPS paradigm with an ISI of 300–500 ms, although it is conceivable that the ERD or ERS induced by the preceding CS may persist at the onset of the subsequent TS, it remains unclear whether PPD is affected by CS-induced oscillation power immediately before TS. PPD is affected by the intensity and pattern of the CS (Lim et al. 2012; Onishi et al. 2016), and it is suggested that the stimulation frequency may influence the duration of ERS induced by median nerve stimulation (Houdayer et al. 2006). Therefore, in this study, we used several types of CS to investigate whether the ERD/ERS magnitude induced by CS affects the subsequent TS response (PPD ratio). We further examined the test–retest reliability of PPD and the alpha, beta, and gamma ERD/ERS induced by somatosensory stimulation using neuromagnetic data. For all analyses, we focused on sensor level SEF waveforms because they directly detect cortical activities.

Some investigators have reported on the relationship between brain-derived neurotrophic factor (BDNF), which is involved in brain development, as well as neuronal plasticity and PPD (Manning and van den Buuse 2013; Naumenko et al. 2013; Notaras et al. 2017; van den Buuse et al. 2017). A single nucleotide polymorphism producing a valine-to-methionine substitution at codon 66 (Val66Met) in the human BDNF gene results in reduced BDNF release to cortical neurons, and is associated with cortical morphology and memory (Egan et al. 2003; Figurov et al. 1996; Hashimoto et al. 2016; Huang et al. 2014; Liu et al. 2014). It has been reported that Met carriers, including Val66Met heterozygotes and Met66Met homozygotes, have reduced use-dependent neural plasticity (Cirillo et al. 2012; Kleim et al. 2006), and that BDNF acutely reduces postsynaptic GABAa receptor-mediated currents (Mizoguchi et al. 2003; Tanaka et al. 1997). Interestingly, the relationship between BDNF gene polymorphisms and PPD has been reported in mice, and BDNF heterozygous mutant mice show reduced PPD (Manning and van den Buuse 2013). In addition, Notaras et al. (2017) recently reported that human BDNF gene polymorphisms are associated with PPD induced by auditory stimulation; the Val66Met allele showed significantly reduced PPD compared with the Val66Val allele (Notaras et al. 2017). Therefore, as a supplemental analysis, we assessed whether the BDNF genotype influences variability of the PPD ratio elicited by somatosensory stimulation.

## Materials and methods

### Participants

Nineteen healthy volunteers (age, 20–35 years; mean ± standard deviation, 23.6 ± 4.2 years; three females) participated in this study. A sample size was calculated using a sample size calculating software (G*Power 3.1.9.2, Germany), and the parameters were set at α = 0.05, power = 0.8, effect size = 0.25, number of measurements = 6, correlation among repeated measures = 0, and nonsphericity correction = 0.2. No subjects were taking medication or had a history of physical or neurological disorders. All subjects gave their written informed consent. The study conformed to the Declaration of Helsinki and was approved by the ethics committee at the Niigata University of Health and Welfare. Twelve out of the 19 (age, 20–27 years; mean ± standard deviation, 22.7 ± 2.3 years; two females) participated in the second measurement to assess the test–retest reliability.

### PPD Paradigm

**Fig. 1:**
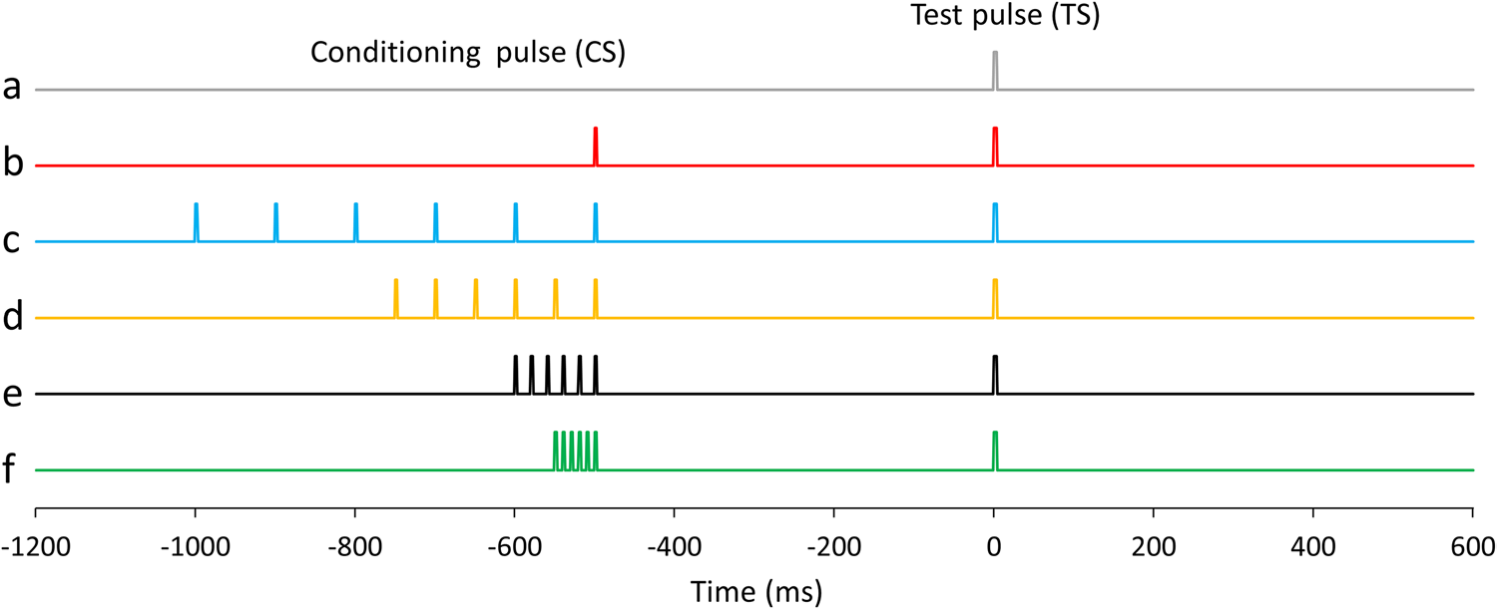
Schematic representation of the electrical stimulation in the PPS paradigm. **a** Single pulse as a test stimulation (TS; condition_a, TS_alone), **b** single pulse as a conditioning stimulation (CS) 500 ms before the TS (condition_b; PPS), **c** six pulse train at 10 Hz as the CS 500 ms before the TS (condition_c, 10 Hz train), **d** six pulse train at 20 Hz as the CS 500 ms before the TS (condition_d, 20 Hz train), **e** six pulse train at 50 Hz as the CS 500 ms before the TS (condition_e, 50 Hz train), and **f** six pulse train at 100 Hz as the CS 500 ms before the TS (condition_f, 100 Hz train). The inter-stimulus interval between the last pulse of the CS and TS in condition_b, _c, _d, _e, and _f was set to 500 ms. Conditions were presented in a pseudorandom order with an inter-TS-TS interval ranging from 4500 to 5500 ms

Electrical stimulation was delivered to the right median nerve using a felt-tip bipolar electrode placed on the wrist with a pulse duration of 0.2 ms (SEN-8203; Nihon Kohden, Tokyo, Japan). The intensity of the electrical stimulation was set at 90% of the motor threshold (MT). The MT was defined as the intensity at which muscle contraction was slightly elicited following a 100 Hz electrical stimulation. Fig. 1 is a schematic representation of the electrical stimulation in this study. We used six stimulation sets: (a) a single pulse as the TS (TS_alone), (b) a single pulse as the CS 500 ms before the TS (PPS), (c) six pulses at 10 Hz as the CS 500 ms before the TS (10 Hz train), (d) six pulses at 20 Hz as the CS 500 ms before the TS (20 Hz train), (e) six pulses at 50 Hz as the CS 500 ms before the TS (50 Hz train), and (f) six pulses at 100 Hz as the CS 500 ms before the TS (100 Hz train). Conditions, including TS_alone, were presented in a pseudorandom order with an inter-TS–TS interval ranging from 4500 to 5500 ms. Eighty or more TSs were delivered for each condition, and MEG measurements took about 40–60 min per subject.

### MEG Recordings

Subjects were comfortably seated in a magnetically shielded room (Tokin Ltd., Sendai, Japan) with their heads firmly positioned inside a 306-ch whole-head MEG system (Vectorview, Elekta, Helsinki, Finland). This device contains 102 identical triple sensors, each housing two orthogonal planar gradiometers and a magnetometer; a configuration of gradiometers that specifically detects signals just above the source current. Continuous MEG signals were sampled at 1000 Hz using a band-pass filter ranging between 0.03 and 330 Hz. Prior to MEG measurement, three anatomical fiducial points (the nasion and bilateral preauricular points) and four indicator coils on the scalp were digitized using a three-dimensional digitizer (Fastraktm; Polhemus, Colchester, VT, USA). Because cortical activity is modulated by the circadian clock (Wilson et al. 2014), all MEG measurements were performed on a Friday afternoon to minimize the influence of circadian rhythms. The test–retest interval for MEG measurements was at least two weeks (range 2–24 weeks; mean ± standard deviation, 8.4 ± 7.1 weeks).

### MEG Analysis

The signal space separation (SSS) method, which separates brain-related and external interference signals, was first applied to reduce environmental and biological noise (MaxFilter software 2.2; Elekta). SSS efficiently separates brain signals from external disturbances based on the fundamental properties of magnetic fields (Taulu et al. 2004; Taulu and Simola 2006).

SEF signals were obtained 1200 ms before, and 800 ms after, the onset of the TS. The band-pass filter was set to between 0.2 and 100 Hz. The averages of more than 80 epochs for SEF in each condition were obtained separately using BESA Research 6.1 software (BESA GmbH, Gräfelfing, Germany). To analyze sensor-level cortical activity, we used the SEF waveforms detected by the gradiometer with the largest response above the primary sensorimotor area in the contralateral hemisphere to the stimulation. To calculate SEF deflections, the 200 ms periods between 1200 and 1000 ms before TS was used as the baseline, and the amplitudes and latencies of the most prominent SEF deflections at each time point (from 0 to 300 ms after the TS) were measured.

As a supplemental analysis, vector sum signals from each pair of gradiometers were calculated by squaring the signals from the two planar-type gradiometers at each sensor’s location, summing the squared signals, and then calculating the square root of the sum (RSS) (Kida et al. 2006, 2007). The PPD ratio was calculated using the following formula: [N20m, P35m, or P60m amplitudes evoked by each TS with CS / N20m, P35m, or P60m evoked by TS_alone] × 100.

A time–frequency analysis was performed for frequencies between 5 and 70 Hz, and latencies between 1200 before and 800 ms after the onset of the TS, in steps of 2.5 Hz and 20 ms respectively, were determined using BESA software. These corresponded to a time–frequency resolution of ± 31.5 ms and ± 3.54 Hz (50% power drop of the FIR filter). In this study, we focused on the alpha band (10.0–12.5 Hz), beta band (15.0–30.0 Hz), and gamma band (32.5–70.0) changes in power between 300 and 500 ms after TS_alone to analyze the relationship between ERD/ERS following median nerve stimulation and the PPD ratio for each condition. In addition, to analyze the effects of ERD/ERF immediately before each TS on the subsequent TS response (PPD ratio), we also calculated the CS-induced oscillatory activity between 300 and 460 ms after the CS as the ERD/ERS immediately before the TS. For each time–frequency bin we used the percentage change in power relative to the mean power in the baseline period between 1200 and 1000 ms before the TS. The temporal spectral evolution method (TES) (Salmelin and Hari 1994) was used to quantify the modulation of rhythmic activity using BESA software.

### DNA Amplification and Genotyping of the Val66Met Polymorphism

Sequences for the design of the genotyping assay were obtained from the SNP database (BDNF-rs6265) of the National Center for Biotechnology Information. DNA was extracted from whole-blood samples using a NucleoSpin Blood Quickpure kit (Macherey–Nagel, Düren, Germany). Samples were genotyped by TaqMan allelic discrimination real-time PCR with CFX connect (Bio-Rad Laboratories, California, USA). Reactions were performed in duplicate using the Kapa Probe Fast qPCR Kit Master Mix (2X) Universal (Kapa Biosystems, Wilmington, MA, USA). We used the following forward and reverse primers: 5′-TGACATCATTGGCTGACACT-3′; 5′-CAAAGGCACTTGACTACTGAG-3′. The probe sequences were FAM-TCGAACACGTGATAGAAGAGCTGTT-BHQ for probe G and HEX-CGAACACATGATAGAAGAGCTGTTGG-BHQ for probe A. The primers and probes were synthesized by Nihon Gene Research Laboratories (Miyagi, Japan). The PCR reaction was conducted in a 20 µL reaction mixture containing 10 µL of PCR Master Mix, 1 µL of each of the two primers and probes, and 6 µL of DNA and DNase-free water. Amplification was carried out by CTF connect under the following conditions: 95 °C for 3 min, followed by 40 cycles at 95 °C for 3 s, 62 °C for 20 s, and 72 °C for 1 s. For each cycle, the fluorescent signal from the HEX- or FAM-labeled probes was determined. The discrimination of genotypes was conducted with Bio-Rad CFX manager 3.1 software.

### Statistical Analysis

Data are expressed as means ± standard error of the mean (SEM). Statistical analyses were performed using IBM SPSS Statistics software 24 (IBM SPSS, Armonk, NY, USA). The latencies for SEF deflections and the amplitude for RSS waveforms were statistically analyzed using a one-way repeated measures ANOVA. The sphericity of the data was analyzed using Mauchly’s test, and Greenhouse-Geisser-corrected significance values were used when sphericity was lacking. When the ANOVA revealed significant differences, Bonferroni correction was used for multiple comparisons, and the percentage of amplitude were statistically analyzed using the Friedman repeated measures ANOVA and Wilcoxon signed rank test. Moreover, Pearson’s product-moment correlation coefficients were used to evaluate the relationship between the PPD ratio of each SEF deflection and changes to each frequency power level. When a significant correlation was found between a PPD ratio and frequency change in power, student’s *t*-test with Bonferroni correction was used to compare the PPD ratio between the ERD and ERS groups. In addition, we calculated the type 1-1 intra-class correlation (ICC) for test–retest reliability, and ICC values were classified as poor (< 0.40), fair (0.41–0.60), good (0.61–0.75), or excellent (> 0.75) (Cicchetti and Sparrow 1981). For all analyses, a *p*-value of < 0.05 was considered indicative of statistical significance.

## Results

### PPD

SEFs were successfully recorded from all subjects. Prominent deflections were observed approximately 20 ms (N20m), 35 ms (P35m), and 60 ms (P60m) after stimulation; however, the P35m deflection was not observed in one subject. There were no significant differences in peak latencies for N20m [F (5, 90) = 0.74, *p* = 0.596, partial η^2^ = 0.039], P35m [F (2.482, 42.201) = 0.793, *p* = 0.484, partial η^2^ = 0.045], or P60m [F (5, 90) = 1.587, *p* = 0.172, partial η^2^ = 0.081] for each condition (Table 1).

**Table 1:** Peak latencies at N20m, P35m, and P60m deflections for each condition

	N20m	P35m	P60m
Condition a (single)	21.2 ± 0.3	34.0 ± 0.9	60.1 ± 2.5
Condition b (PPS)	21.2 ± 0.3	33.9 ± 0.8	61.1 ± 2.7
Condition c (10 Hz train)	21.2 ± 0.3	34.5 ± 1.1	56.1 ± 2.5
Condition d (20 Hz train)	21.2 ± 0.3	34.4 ± 1.1	61.7 ± 2.3
Condition e (50 Hz train)	21.2 ± 0.3	34.1 ± 0.9	60.7 ± 2.6
Condition f (100 Hz train)	21.2 ± 0.3	34.2 ± 1.0	60.1 ± 2.4

Mean ± SEM (ms)

**Fig. 2:**
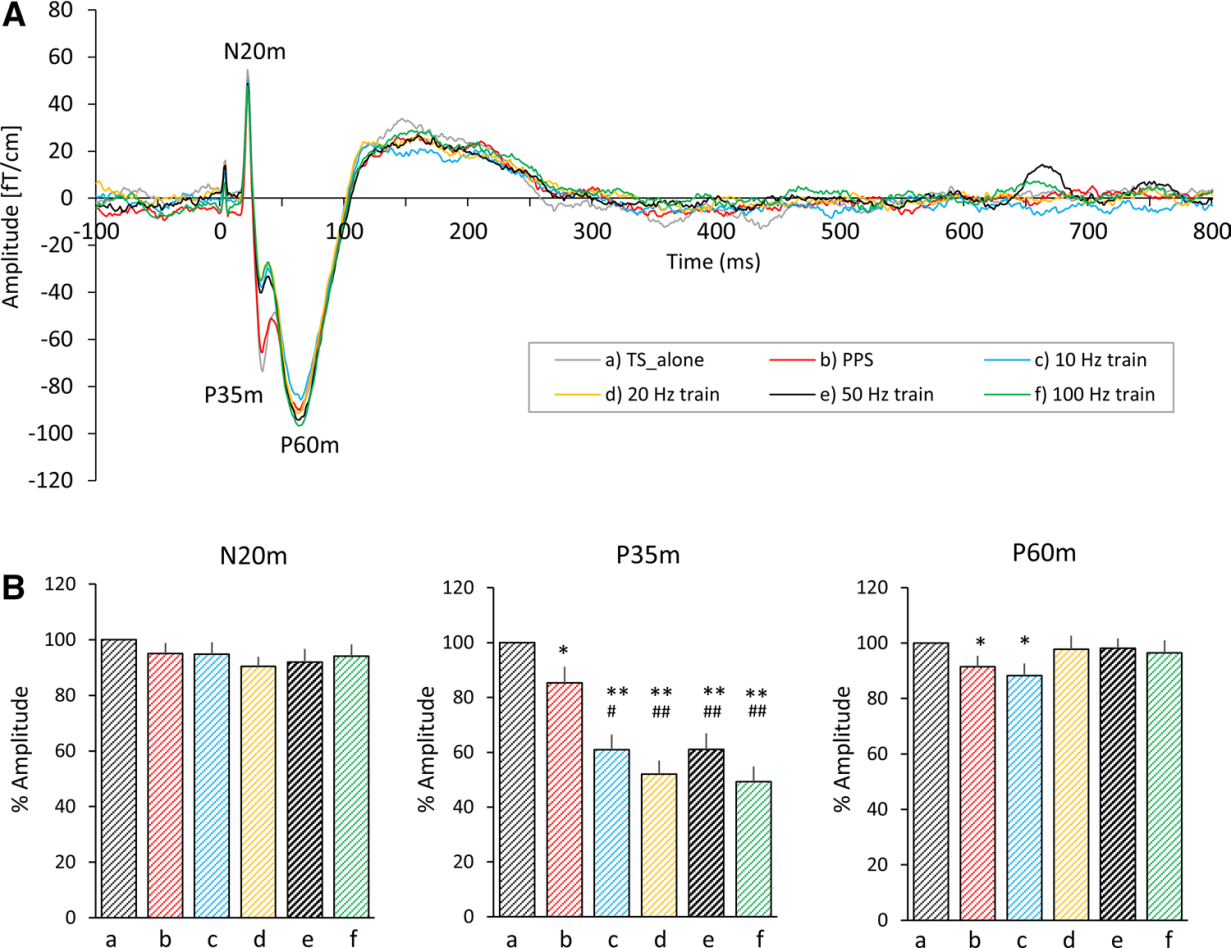
Grand averaged SEF waveforms and mean amplitudes of prominent SEF deflections. **A** The time-course of the grand averaged SEF waveforms from 100 ms before to 800 ms after a test stimulus elicited by all conditions are superimposed. The gray, red, blue, orange, black, and green lines indicate the SEF waveforms elicited by condition_a, _b, _c, _d, _e, and _f respectively. **B** The mean SEF amplitude at N20m, P35m, and P60m. a, b, c, d, e, and f under each bar graph indicate condition_a, _b, _c, _d, _e, and _f respectively. The error bars indicate the standard error of the mean (SEM). *p < 0.05 (vs. condition_a), **p < 0.01 (vs. condition_a), ##*p* < 0.01 (vs. condition_b)

The grand averaged SEF waveforms elicited for each condition 100 ms before and 800 ms after the TS were superimposed in Fig. 2a to compare the time-course of the SEF waveforms elicited by each condition. Friedman test revealed that there was no significant difference in percentage of amplitude at N20m (*p* = 0.203), but there were statistical differences in amplitude at P35m (*p* = 3.34 × 10^−9^) and at P60m (*p* = 0.001). Post hoc testing indicated that the percentage of amplitude at P35m elicited by condition_b was significantly smaller than that elicited by condition_a (*p* = 0.018), and those elicited by condition_c, _d, _e, and _f were significantly smaller than that elicited by condition_a (condition_c, *p* = 7.38 × 10^−4^; condition_d, *p* = 2.33 × 10^−4^; condition_e, *p* = 5.36 × 10^−4^; condition_f, *p* = 1.96 × 10^−4^ respectively) and condition_b (condition_c, *p* = 0.012; condition_d, *p* = 0.0012; condition_e, *p* = 0.005; condition_f, *p* = 5.36 × 10^−4^). The percentage of amplitude at P60m elicited by condition_b and _c was significantly smaller than that elicited by condition_a (condition_b, *p* = 0.022; condition_c, *p* = 0.014, Fig. 2b). The grand averaged RSS waveforms elicited by each condition are superimposed in Supplementary Fig. 1A, and mean RSS amplitudes at N20m, P35m, and P60m are shown in Supplementary Fig. 1B. The results for the RSS amplitudes were almost same as the results for the SEF waveforms.

### Time Frequency Changes in Power Induced by TS_alone and the PPD Ratio

**Fig. 3:**
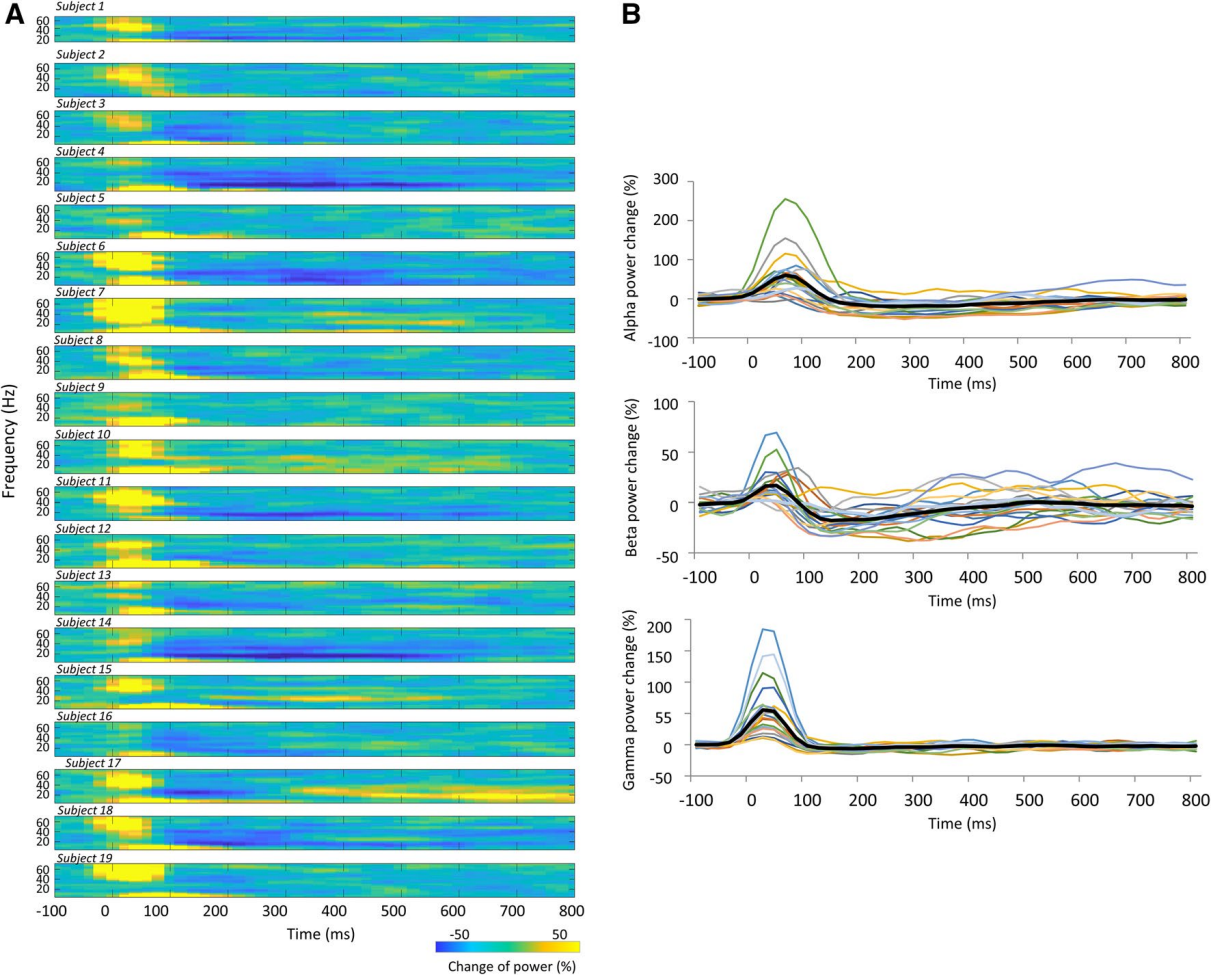
Time-course of frequency changes in power induced by condition_a (TS_alone). **A** Time frequency maps of signal power changes from 100 ms before to 800 ms after the test stimulation in all subjects. **B** Time-course of signal changes in power for each frequency from 100 ms before to 800 ms after the test stimulation band in all subjects

**Fig. 4:**
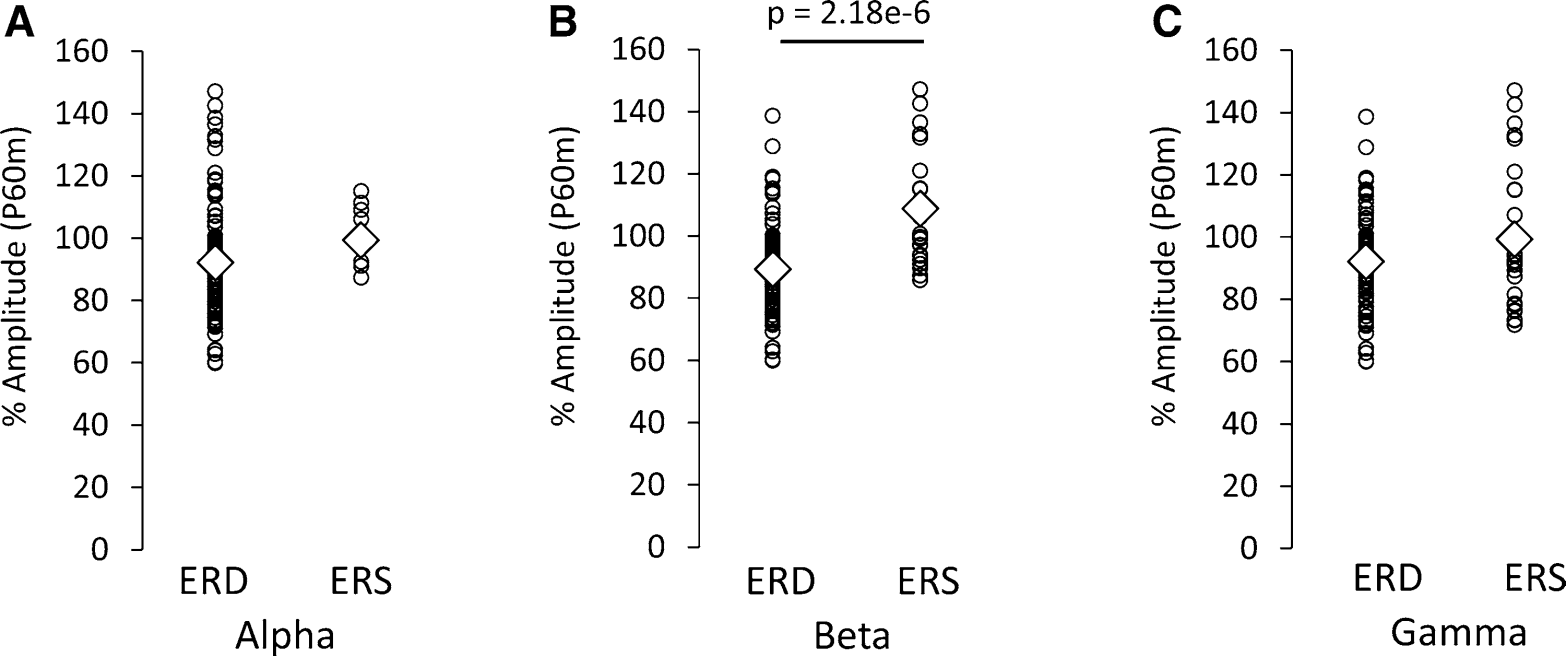
Comparison of the P60m_PPD ratio induced under all conditions with conditioning stimulation (condition_b, _c, _d, _e, and _f) between the ERS and ERD groups induced with test stimulation under condition_a. Relationships between the P60m_PPD ratio and the **A** alpha, **B** beta, and **C** gamma bands. The P60m_PPD ratio was significantly smaller for the group in which beta ERD was observed than for the group in which beta ERS was observed. There were no significant differences in the P60m_PPD ratio between the ERD and ERS groups in the alpha and gamma bands

Time frequency maps of signal power changes and the time-course of power changes in the alpha, beta, and gamma band 100 ms before and 800 ms after TS for condition_a (TS_alone) are summarized in Fig. 3. We observed that the ERD/ERS between 300 and 500 ms after the TS, and the ERD/ERS magnitude, differed between subjects. Table 2 shows the Pearson’s product-moment correlation coefficient (r) for the relationship between the PPD ratio at N20m, P35m, and P60m evoked by each PPS paradigm (condition_b, _c, _d, _e, and _f) and each frequency (alpha, beta, and gamma) change in power between 300 and 500 ms after TS_alone (condition_a). Statistically significant positive correlations were only observed between the P60m_PPD ratio and beta power changes for each PPS paradigm. Supplementary Fig. 2 shows the correlation between the pooled PPD ratio data at N20m, P35m, and P60m that was induced by all conditions with the CS (condition_b, _c, _d, _e, and _f), and alpha, beta, and gamma power changes between 300 and 500 ms after condition_a (TS_alone). Statistically significant positive correlations were observed between the alpha, beta, and gamma power changes and the P60m_PPD ratio. The Pearson’s correlation coefficient (r) and the corresponding *p* value for these correlations were r = 0.28 for alpha power changes (*p* = 0.007), r = 0.53 for beta power changes (*p* = 3.20 × 10^−8^), and r = 0.33 for gamma power changes (*p* = 9.37 × 10^−4^). No significant correlations were observed between each frequency power change and the PPD ratio at N20m or P35m. The P60m_PPD ratio was significantly smaller for the group in which beta ERD was observed (89.3 ± 1.9%, n = 70) than for the group in which beta ERS was observed (108.7 ± 3.6%, n = 25; *p* = 2.18 × 10^−6^, d = 1.18). No significant differences were observed in the P60m_PPD ratio between the alpha ERD group (93.7 ± 2.1%, n = 85) and the alpha ERS group (100.6 ± 3.0%, n = 10; *p* = 0.267, d = 0.37), or between the gamma ERD group (92.2 ± 2.1%, n = 65) and the gamma ERS group (99.3 ± 4.0%, n = 30; *p* = 0.081, d = 0.36; Fig. 4).

**Table 2:** Pearson’s product-moment correlation coefficients (r) between the PPD ratio at N20m, P35m, and P60m induced by each PPS paradigm (condition_b, _c, _d, _e, and _f) and each frequency (alpha, beta, and gamma) change in power between 300 and 500 ms after test stimulation under condition_a

	α	β	γ
Condition b (PPS)			
N20m	0.07	0.12	− 0.33
P35m	0.23	0.10	0.20
P60m	0.38	0.50*	0.44
Condition c (10 Hz train)			
N20m	0.23	0.09	0.31
P35m	0.10	0.11	0.11
P60m	0.28	0.54*	0.27
Condition d (20 Hz train)			
N20m	−0.04	0.06	− 0.18
P35m	−0.15	− 0.09	− 0.01
P60m	0.23	0.55*	0.31
Condition e (50 Hz train)			
N20m	− 0.17	− 0.25	− 0.26
P35m	0.05	0.16	0.13
P60m	0.33	0.55*	0.39
Condition f (100 Hz train)			
N20m	0.13	0.09	− 0.12
P35m	− 0.23	− 0.01	− 0.03
P60m	0.23	0.59**	0.33
All			
N20m	0.04	0.01	− 0.11
P35m	0.01	0.05	0.08
P60m	0.28**	0.53**	0.33**

*p < 0.05; **p < 0.01

### CS-Induced Time Frequency Changes in Power and PPD Ratio

**Fig. 5:**
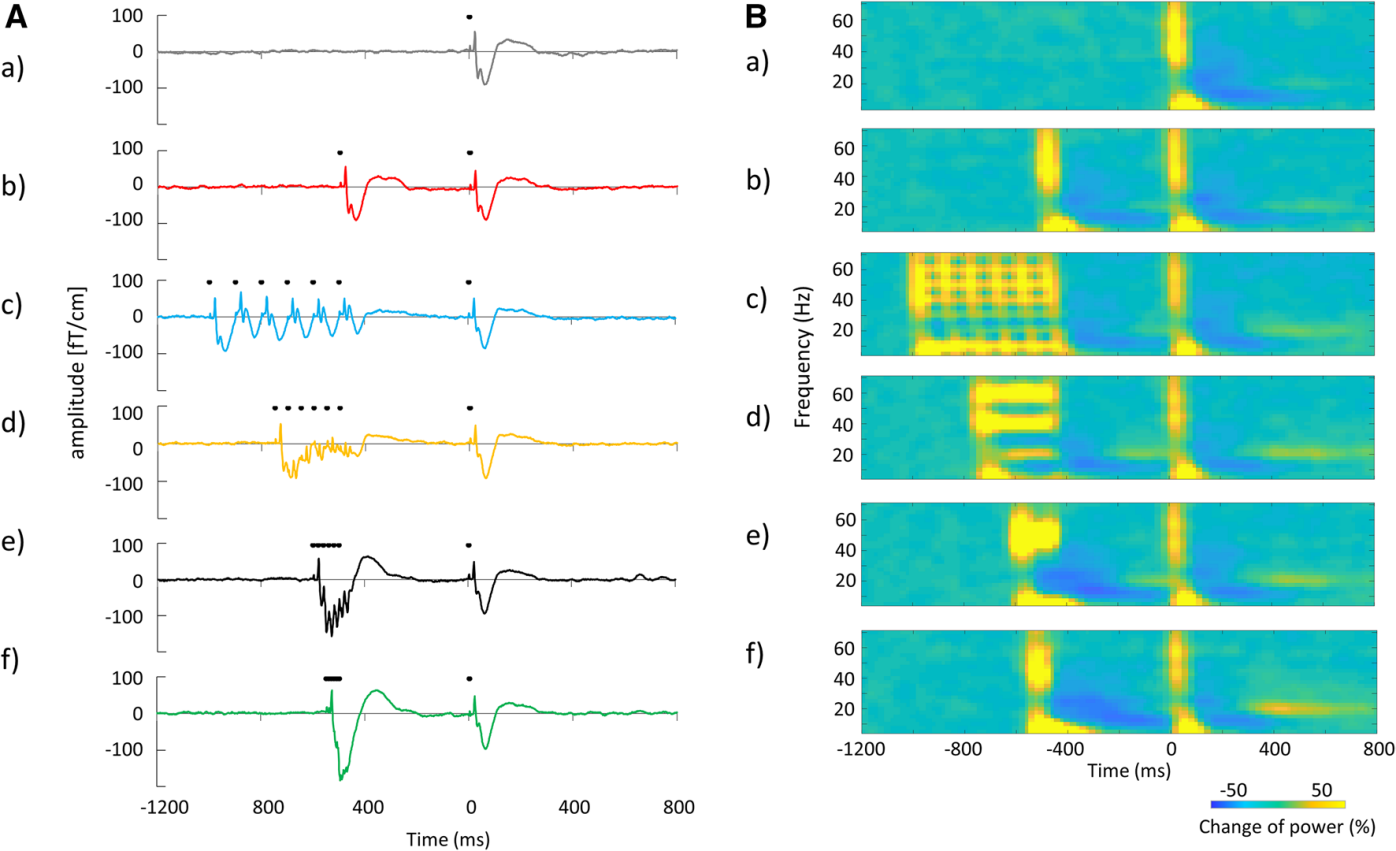
**A** Grand averaged SEF waveforms and **B** time frequency maps of changes in power under each condition from 1200 ms before to 800 ms after the test stimulation. (*a*–*f*) indicate condition_a, _b, _c, _d, _e, and _f respectively. The black dots above the averaged SEF waveforms indicated the points of electrical stimulation

**Fig. 6:**
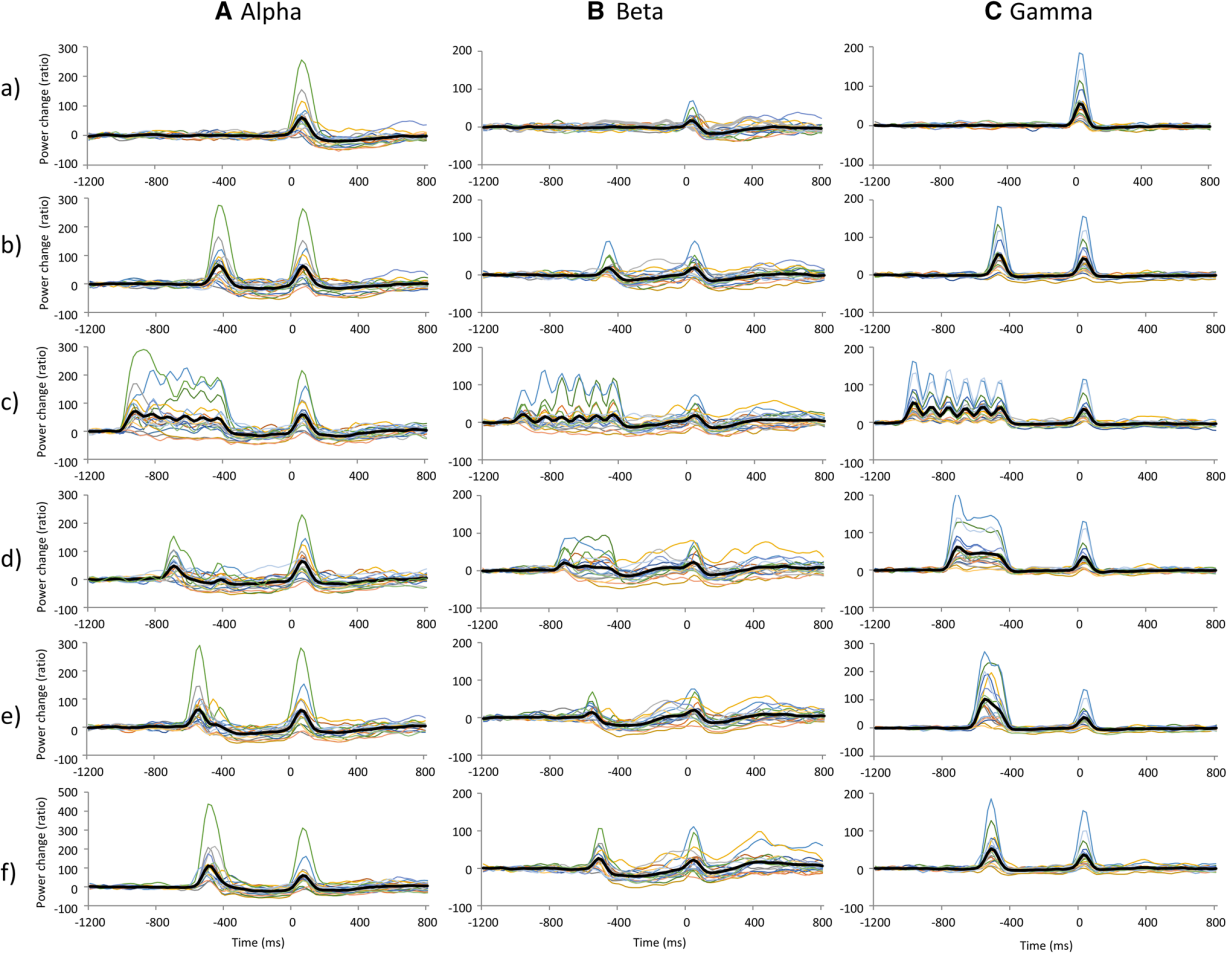
Time-course of signal changes in power for each frequency band from 1200 ms before to 800 ms after the test stimulation in all subjects. **A** Alpha, **B** beta, and **C** gamma band changes in power. (*a*–*f*) indicate condition_a, _b, _c, _d, _e, and _f respectively. The thick black lines on each graph indicate the mean of the data and the thin lines indicate individual data

**Fig. 7:**
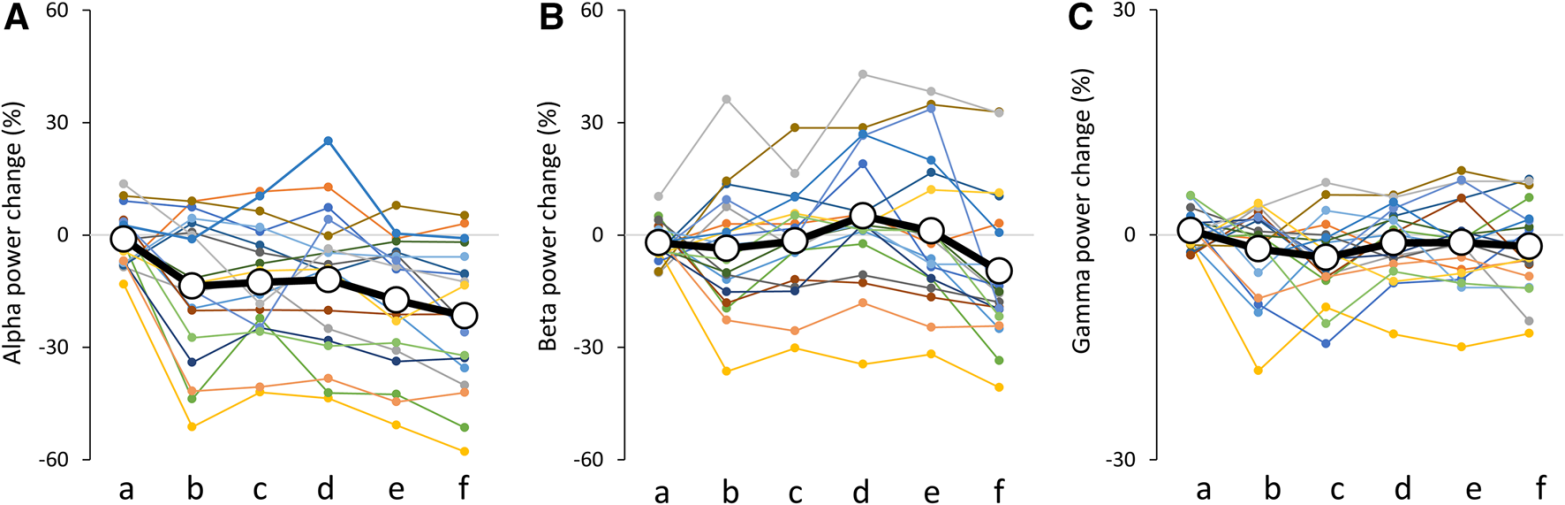
Scatter plots showing the mean and individual change in frequency power from 300 to 40 ms before test stimulation. **A** Alpha, **B** beta, and **C** gamma band changes in power. a, b, c, d, e, and f under each graph indicate condition_a, _b, _c, _d, _e, and _f respectively. The thick black line on each graph indicates the mean of the data and the thin lines indicate individual data

**Fig. 8:**
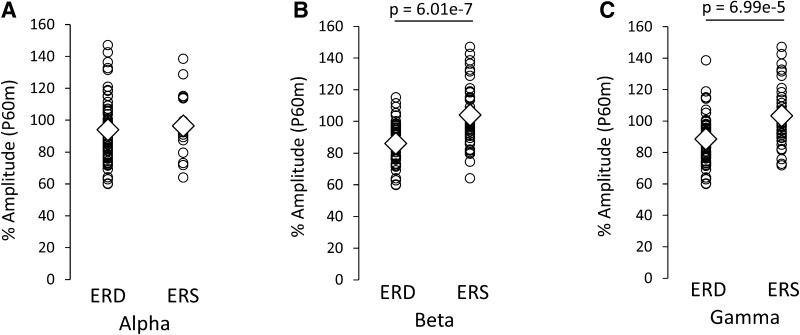
Comparison of the P60m_PPD ratio induced under all conditions with conditioning stimulation (condition_b, _c, _d, _e, and _f) between the ERS and ERD groups immediately before test stimulation, following each conditioning stimulation (condition_b, _c, _d, _e, and _f). **A** Alpha, **B** beta, and **C** gamma band changes in power. The PPD ratio at P60m was significantly smaller for the beta and gamma ERD groups than those for the beta and gamma ERS groups. There were no significant differences in the PPD ratio at P60m between the alpha ERD and alpha ERS groups

The grand averaged SEF waveforms and the time frequency maps of signal changes in power 1200 ms before and 800 ms after TS for each condition are summarized in Fig. 5. The time-course of signal changes in power for each frequency band measured in all subjects is shown in Fig. 6. Mean alpha and beta power increased immediately after the CS onset. This augmentation was followed by alpha ERD and beta ERD; however, a transitory mean alpha ERD was sustained immediately before the TS, while the mean beta ERD almost returned to baseline before the TS. Individual data showed large variability in alpha and beta band changes in power, particularly in the beta band. Although mean gamma band changes in power were relatively small compared with alpha and beta band changes for all conditions, individual gamma band changes in power before the TS showed some variability (Fig. 7). Table 3 shows the Pearson’s product-moment correlation coefficient (r) for the relationship between the PPD ratio at N20m, P35m, and P60m evoked by each PPS paradigm (condition_b, _c, _d, _e, and _f) and each frequency (alpha, beta, and gamma) change in power between 300 and 460 ms after the CS (condition_b, _c, _d, _e, and _f). Statistically significant positive correlations were observed between the P60m_PPD ratio and alpha, beta, gamma power changes for several conditions, and between P35m_PPD ratio and alpha power changes for condition c. Supplementary Fig. 3 shows the correlation between the pooled data of the PPD ratio for each SEF deflection (N20m, P35m, and P60m) and the pooled data of changes in power for each frequency band (alpha, beta, and gamma) between 300 and 460 ms after the CS. Statistically significant positive correlations were observed between alpha, beta, and gamma power changes and the P60m_PPD ratio. The Pearson’s correlation coefficients (r) and the corresponding *p*-values for these correlations were r = 0.34 for alpha power (*p* = 2.22 × 10^−4^), r = 0.51 for beta power (*p* = 5.68 × 10^−9^), and r = 0.42 for gamma power (*p* = 3.15 × 10^−6^). No significant correlations were observed between each frequency power change and the PPD ratio at N20m or P35m. The P60m_PPD ratio was significantly smaller for the group in which beta ERD was observed (86.1% ± 1.9%, n = 51) compared with that for the group in which beta ERS was observed (104.1 ± 3.0%, n = 44; *p* = 6.01 × 10^−7^, d = 1.1). Moreover, the P60m_PPD ratio was significantly smaller for the gamma ERD group (88.5 ± 2.1%, n = 57) than for the gamma ERS group (103.4 ± 3.1%, n = 38; *p* = 6.99 × 10^−5^, d = 0.87). There were no significant differences in the P60m_PPD ratio between the alpha ERD group (93.9 ± 2.1%, n = 76) and the alpha ERS group (96.4 ± 19.0%, n = 19; *p* = 0.60, d = 0.03; Fig. 8).

**Table 3:** Pearson’s product-moment correlation coefficients (r) between the PPD ratio at N20m, P35m, and P60m induced by each PPS paradigm (condition_b, _c, _d, _e, and _f), and each frequency (alpha, beta, and gamma) change in power immediately before test stimulation, following each conditioning stimulation (condition_b, _c, _d, _e, and _f)

	α	β	γ
Condition b (PPS)			
N20m	− 0.02	0.17	− 0.16
P35m	0.16	0.04	0.31
P60m	0.32	0.56*	0.51*
Condition c (10 Hz train)			
N20m	0.05	0.09	0.05
P35m	0.48*	0.14	0.32
P60m	0.27	0.40	0.25
Condition d (20 Hz train)			
N20m	− 0.11	− 0.03	− 0.32
P35m	0.07	− 0.14	0.02
P60m	0.46*	0.53*	0.40
Condition e (50 Hz train)			
N20m	− 0.19	− 0.29	− 0.37
P35m	0.06	0.08	− 0.14
P60m	0.44	0.52*	0.27
Condition f (100 Hz train)			
N20m	0.11	0.06	0.16
P35m	− 0.04	0.05	− 0.03
P60m	0.30	0.54*	0.52*
All			
N20m	− 0.04	− 0.04	− 0.14
P35m	0.15	0.03	0.06
P60m	0.34**	0.51**	0.42**

*p < 0.05; **p < 0.01

### BDNF Gene Polymorphism

Nineteen subjects were genotyped as follows: four participants (21.0%) were found to be homozygotes for Val66Val, 12 (63.2%) were Val66Met heterozygotes, and three (15.8%) were homozygotes for Met66Met (Table 4). We could find no differences in PPD ratio among BDNF gene polymorphisms. However, because there were few subjects with Val66Val and Met66Met allele, it was difficult to conduct statistical analyses to evaluate the influence of BDNF gene polymorphisms on the PPD ratio for each component.

**Table 4:** Summary of BDNF genotype, PPD ratio for each SEF component, and each frequency change in power in an individual participant

Subject	BDNF genotype	PPD ratio (% amplitude)			Frequency power change (%)		
		N20m	P35m	P60m	α	β	γ
1	Val/Val	111.4	46.4	69.3	− 15.9	− 4.8	− 1.0
2	Val/Met	72.3	140.8	128.8	11.6	3.0	1.4
3	Met/Met	88.7	65.5	91.5	− 11.8	− 3.8	− 5.3
4	Val/Met	53.3	60.1	80.5	− 41.9	− 30.2	− 9.7
5	Val/Met	116.8	35.3	90.9	0.9	1.9	− 14.5
6	Val/Met	106.1	62.1	77.1	− 22.2	− 4.1	− 6.1
7	Val/Val	91.9	60.1	115.1	− 2.7	10.3	− 4.6
8	Met/Met	84.8	53.9	93.4	− 20.0	− 11.9	− 5.6
9	Val/Met	121.9	76.7	76.5	− 4.7	− 14.0	0.0
10	Val/Met	108.7	54.3	92.4	6.4	28.6	5.3
11	Val/Met	115.9	34.2	95.3	− 24.8	− 15.0	− 3.3
12	Met/Met	85.6	–	78.6	− 7.6	− 1.2	− 0.8
13	Val/Met	110.4	43.4	71.9	2.1	− 1.0	3.2
14	Val/Met	96.0	51.0	60.2	− 40.6	− 25.7	− 5.6
15	Val/Met	90.8	57.8	121.0	− 18.4	16.4	6.9
16	Val/Val	89.9	65.9	99.7	− 9.5	5.7	− 2.2
17	Val/Met	102.6	50.3	97.1	− 24.8	− 0.7	− 3.7
18	Val/Val	95.3	44.1	74.5	− 25.8	5.2	− 11.9
19	Val/Met	58.1	93.5	64.1	10.4	10.0	− 0.4
Mean	Val/Val (n = 4)	97.1	54.2	89.6	− 13.5	4.1	− 4.9
Mean	Val/Met (n = 12)	96.1	63.3	88.0	− 12.2	− 2.6	− 2.2
Mean	Met/Met (n = 3)	86.3	59.7*	87.9	− 13.1	− 5.6	− 3.9
Mean	All (n = 19)	94.8	60.9	88.3	− 12.6	− 1.7	− 3.0

*P35m deflection was not observed in subject 12; therefore, the mean PPD ratio at P35m for the Met66Met allele was calculated for only two subjects

The PPD ratio and frequency power change obtained for condition_c (10 Hz train). 4, 12, and 3 out of the 19 participants showed the Val66Val, Val66Met, and Met66Met alleles respectively

### Test–Retest Reliability

**Fig. 9:**
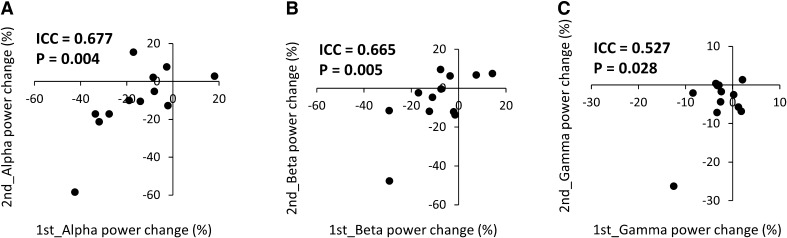
Correlations between the test and retest alpha, beta, and gamma band changes in power induced by test stimulation alone (condition_a). **A** Alpha, **B** beta, and **C** gamma band changes in power

Time frequency maps of signal power changes 100 ms before and 800 ms after TS_alone in all subjects who participated in the two measurements for test–retest reliability are shown in Supplementary Fig. 4. ICC values for the alpha and beta frequency changes in power after TS alone indicated good reliability, while those for gamma power indicated fair reliability (Fig. 9). Table 5 shows the results of the ICC in the PPD ratio at N20m, P35m, P60m, and the alpha, beta, and gamma power changes between 300 and 460 ms after the CS. The reliability of the PPD ratio varied among the N20m, P35m, and P60m measurements. ICC values for the pooled data of N20m_PPD ratio showed no statistically significant correlation (ICC = − 0.045, *p* = 0.634), whereas the P35m_PPD ratio indicated excellent reliability (ICC = 0.757, *p* = 4.44 × 10^−11^). The ICC values for the pooled data of P60m_PPD ratio indicated poor reliability, and this result was statistically significant (ICC = 0.303, *p* = 0.008; Supplementary Fig. 5 A–C). On the other hand, ICC values for the pooled data of alpha, beta, and gamma power changes induced by the CS between 300 and 460 ms after the CS indicated were high: 0.680 for alpha (*p* = 6.56 × 10^−10^), 0.760 for beta (*p* = 4.36 × 10^−13^), and 0.552 for gamma (*p* = 1.76 × 10^−6^) frequency changes in power (Supplementary Fig. 5 D–F).

**Table 5:** Intra-class correlations between the test and retest PPD ratio at N20m, P35m, and P60m, and the alpha, beta, and gamma power changes immediately before test stimulation, following each conditioning stimulation (condition_b, _c, _d, _e, and _f)

	PPD ratio (% amplitude)			ERD/ERS		
	N20m	P35m	P60m	α	β	γ
Condition b (PPS)	0.279	0.897**	0.426	0.636**	0.680**	0.644**
Condition c (10 Hz train)	0.002	0.700**	0.280	0.830**	0.787**	0.414
Condition d (20 Hz train)	− 0.154	0.550*	0.351	0.599**	0.795**	0.518*
Condition e (50 Hz train)	− 0.349	0.761**	0.323	0.750**	0.848**	0.782**
Condition f (100 Hz train)	0.228	0.759**	0.212	0.694**	0.718**	0.473*
All	− 0.045	0.757**	0.303*	0.680**	0.760**	0.552**

*p < 0.05; **p < 0.01

## Discussion

The main findings of this study, which used a somatosensory PPD paradigm with a 500 ms CS–TS interval, are: (1) the P60m_PPD ratio is correlated with beta ERD magnitude after median nerve stimulation; (2) the P60m_PPD ratio is influenced by beta ERD and gamma ERD magnitudes just before the TS; (3) the P35m_PPD is robust and highly reproducible; (4) the P60m_PPD ratio is small compared to the P35m_PPD, and the ICC is poor but statistically significant; and (5) the alpha, beta, and gamma ERD/ERS induced by median nerve stimulation are highly reproducible.

We observed a correlation between the P60m_PPD ratio and beta ERD magnitude following median nerve stimulation. The P60m_PPD ratio was significantly smaller for the beta ERD group than for the beta ERS group. At the cellular level, there are many reports investigating the mechanisms of PPD, and both postsynaptic and presynaptic mechanisms underlying PPD have been proposed. Regarding postsynaptic mechanisms, PPD is thought to be caused by decreased Cl^−^ conductance due to intracellular accumulation of Cl^−^ and extracellular accumulation of K^+^ (McCarren and Alger 1985), as well as a decreased sensitivity of GABAa receptors (Alger 1991). The most widely accepted model for the PPD mechanism is a presynaptic mechanism resulting from the autoinhibition of GABA rerelease due to the activation of presynaptic GABAb receptors, which is followed by a decrease in calcium current at the terminal (Davies et al. 1990; Deisz and Prince 1989; Pearce et al. 1995; Wilcox and Dichter 1994). Most of these studies have been performed in animal models. Therefore, whether these PPD mechanisms can be applied directly to humans is unclear. However, it has been reported that GABA affects PPD in human subjects (Huttunen et al. 2008; Stude et al. 2016). Beta ERD has also been found to be affected by GABA concentration (Hall et al. 2011; Muthukumaraswamy et al. 2013), and Jensen et al. demonstrated that increasing connections from inhibitory interneurons to excitatory pyramidal neurons induces a decrease in beta frequency power using conductance based neuronal-network models (Jensen et al. 2005). This is consistent with the significant correlation between the P60m_PPD ratio and the beta ERD magnitude observed in this study. The variability of GABA concentration between subjects in the adult human brain (Evans et al. 2010; Near et al. 2014) may influence the P60m_PPD variability between subjects in this study. However, because we did not measure GABA concentrations in this study, we intend to perform further investigations to clarify the relationship between GABA concentration in the primary somatosensory cortex and the P60m_PPD ratio.

The magnitude of gamma ERD just before the TS also affected the subsequent P60m amplitude. In this regard, the P60m_PPD ratio was significantly smaller for the gamma ERD group than the gamma ERS group. PPD is attenuated or disappears in patients with schizophrenia (Swerdlow et al. 2008). In these patients gamma frequency power is increased (Flynn et al. 2008; Spencer 2011), and somatosensory evoked potentials decrease when the background gamma frequency power is high (Jones et al. 2014; Kulikova et al. 2012). Therefore, we expected that the gamma frequency power just before TS would modulate the subsequent TS-evoked response, and that the PPD would disappear when gamma ERS was observed just before the TS. Indeed, we confirmed that the gamma frequency power just before the TS affected the subsequent TS-evoked response. We consider this to be one of the factors that cause PPD variability in the somatosensory PPS paradigm.

Many studies have typically focused on the P35m component of SEF to investigate somatosensory gating. However, it has been reported that P35m and P60m have different current sources and different GABA agonist responses (Huttunen et al. 2006, 2008; Onishi et al. 2016; Wikström et al. 1996). Therefore, it is important to clarify the differences in PPD between P35m and P60m for clinical application. Furthermore, the reproducibility of PPD following somatosensory stimulation has not yet been investigated. In order to use PPD as a clinical biomarker, it is necessary to define a reproducible measurement for PPD. In this study, P35m_PPD was robust and highly reproducible: the reproducibility of P60m_PPD was weaker than that of P35m. These results suggest that P35m is a suitable evaluation index for inhibitory function in the somatosensory cortex. However, because P35m_PPD was not related to beta ERD, which is reportedly related to GABA concentration (Cheng et al. 2017), the mechanisms of P35m_PPD need to be elucidated.

In our previous study, we investigated the effect of intensity and ISI on PPD using several types of CS in a PPS paradigm (Onishi et al. 2016). We found that the inhibitory effects of CS on the TS response at P60m lasted for a longer period than that at P35m, and that the PPD at P60m was observed when a three pulse CS was presented with a 250–1000 ms CS–CS interval (Onishi et al. 2016). Therefore, in the previous study, we concluded that the inhibitory effects found for P60m were accumulated. However, the accumulation effect for P60m was not observed with six pulses with a 10–50 ms ISI as the CS in this study. These findings suggest that the ISI of the CS must be greater than 100 ms to cause an accumulation of the inhibitory effect.

It has been reported that BDNF gene polymorphisms influence the PPD ratio under auditory stimulation, and that the PPD is smaller for the Val66Met polymorphism than for the Val66Val polymorphism (Notaras et al. 2017). However, in this study, there were only a few subjects with the Val66Val and Met66Met polymorphism; thus, we could not identify a relationship between the PPD ratio induced by somatosensory stimulation and BDNF gene polymorphisms. The proportion of BDNF gene polymorphisms differs between Caucasian and Japanese populations. The proportion of BDNF gene polymorphisms is 65.1% for Val66Val, 31.5% for Val66Met, and 3.4% for Met66Met in the Caucasian population (Jonsson et al. 2006), and 33.6–35.3% for Val66Val, 48.0–50.7% for Val66Met, and 15.6–17.5% for Met66Met in the Japanese population (Naoe et al. 2007; Tochigi et al. 2006; Watanabe et al. 2006). However, in this study, the frequency of Val66Val was extremely low, while that of Val66Met was high compared to previous studies. Therefore, it was difficult to use statistical analysis to clarify the relationship between PPD ratio and BDNF gene polymorphisms.

We focused on SEF amplitude and cortical oscillation at the sensor level in this study. Source activity is calculated from multiple sensor level activities, yet sensor level waveforms directly detect cortical activity. In other words, before source level analysis, we considered it necessary to evaluate the reproducibility of the PPD ratio and cortical oscillations detected at the sensor level. Although there has been a report on the reproducibility of visually induced cortical oscillation in sensor and source signals (Tan et al. 2016), there have been no reports on the reproducibility of PPD and cortical oscillation evoked by somatosensory stimulation. Thus, our findings should contribute to a better understanding of these processes.

## Conclusion

In this study, we investigated PPD variability and reliability induced by median nerve stimulation using MEG. We found that attenuation of the P35m deflection was robust and that P35m_PPD showed high reliability, even if the CS was presented 500 ms before the TS. On the other hand, although P60m_PPD had poor reliability, the P60m_PPD ratio was significantly correlated with beta ERD/ERS. Moreover, the subjects with beta ERS induced by somatosensory stimulation had small or no P60m_PPD, and beta and gamma ERS/ERD just before the TS affected the PPD ratio. These results indicate that ERD/ERS magnitude influences the variability of PPD for the P60m deflection induced by median nerve stimulation.

## Electronic supplementary material

Below is the link to the electronic supplementary material.

Supplementary Figure 1 Grand averaged RSS waveforms and the mean amplitudes of prominent deflections. (A) Time-courses of the grand averaged RSS waveforms from 20 ms before to 300 ms after the test stimulation elicited by all conditions are superimposed. The gray, red, blue, orange, black, and green lines indicate the RSS waveforms elicited by condition_a, _b, _c, _d, _e, and _f respectively. (B) The mean RSS amplitude at N20m, P35m, and P60m. The a, b, c, d, e, and f under each bar graph indicate condition_a, _b, _c, _d, _e, and _f respectively. The error bars indicate the standard error of the mean (SEM). *p < 0.05 (vs. condition_a), **p < 0.01 (vs. condition_a), ##*p* < 0.01 (vs. condition_b). (TIF 1811 KB)

Supplementary Figure 2 Pearson’s product-moment correlation coefficients (r) between the PPD ratio at N20m, P35m, and P60m induced by all conditions with conditioning stimulation (condition_b, _c, _d, _e, and _f), and changes in power for each frequency band (alpha, beta, and gamma) induced by test stimulation under condition_a. (A) The relationship between alpha power changes and the PPD ratio at N20m, (B) beta power changes, and the PPD ratio at N20m, (C) gamma power changes and the PPD ratio at N20m, (D) alpha power changes and the PPD ratio at P35m, (E) beta power changes and the PPD ratio at P35m, (F) gamma power changes and the PPD ratio at P35m, (G) alpha power changes and the PPD ratio at P60m, (H) beta power changes and the PPD ratio at P60m, and (I) gamma power changes and the PPD ratio at P60m. Statistically significant positive correlations were observed between the PPD ratio at P60m and alpha, beta, and gamma power changes, whereas no significant correlation was observed at N20m or P35m. The red, blue, orange, black, and green dots indicate the data elicited by conditions_b, _c, _d, _e, and _f respectively. (TIF 1396 KB)

Supplementary Figure 3 Pearson’s product-moment correlation coefficients (r) between the PPD ratio at N20m, P35m, and P60m induced under all conditions with conditioning stimulation (condition_b, _c, _d, _e, and _f), and changes in power for each frequency band immediately before test stimulation, following each conditioning stimulation (condition_b, _c, _d, _e, and _f). A) N20m vs. alpha, B) N20m vs. beta, C) N20m vs. gamma, D) P35m vs. alpha, E) P35m vs. beta, F) P35m vs. gamma, G) P60m vs. alpha, H) P60m vs. beta, and I) P60m vs. gamma. Positive correlations were observed between the PPD ratio at P60m and alpha, beta, and gamma power changes, while no significant correlation was observed at N20m or P35m. The red, blue, orange, black, and green dots indicate the data elicited under condition_b, _c, _d, _e, and _f respectively. (TIF 1413 KB)

Supplementary Figure 4 Time frequency maps of signal power changes induced by condition_a (TS_alone) from 100 ms before to 800 ms after the test stimulation in all subjects. The left column shows the maps induced by the first measurement, and the right column shows the maps induced by the second measurement in the same subject. (TIF 1982 KB)

Supplementary Figure 5 Pearson’s product-moment correlation coefficients (r) between the test and retest PPD ratio at N20m, P35m, and P60m and the alpha, beta, and gamma power changes immediately before test stimulation, following each conditioning stimulation (condition_b, _c, _d, _e, and _f). A) N20m, B) P35m, C) P60m, D) alpha power changes, E) beta power changes, and F) gamma power changes. The red, blue, orange, black, and green dots indicate the data obtained for condition_b, _c, _d, _e, and _f respectively. Significant positive correlation in the PPD ratio between the test and retest measurements was noted; ICC values were excellent for P35m, but poor for P60m. Significant positive correlations in the alpha, beta, and gamma power changes between test and retest measurements were noted; the ICC values were good for alpha, excellent for beta, and fair for gamma power changes. (TIF 1198 KB)

## Abbreviations

BDNF: Brain-derived neurotrophic factor
CS: Conditioning stimulation
ERD: Event-related desynchronization
ERS: Event-related synchronization
GABA: γ-Aminobutyric acid
ICC: Intra-class correlation coefficient
ISI: Inter-stimulus interval
MEG: Magnetoencephalography
MT: Motor threshold
PPD: Paired pulse depression
PPS: Paired pulse stimulation
RSS: Square root of the sum
SEF: Somatosensory evoked magnetic field
SSS: Signal space separation
TS: Test stimulation
