# Supplementary figures and images for: Variability and Reliability of Paired-Pulse Depression and Cortical Oscillation Induced by Median Nerve Stimulation
Source: Brain Topogr. 2018 May 8;31(5):780–94. doi: 10.1007/s10548-018-0648-5 (PMC6097743; doi:10.1007/s10548-018-0648-5)

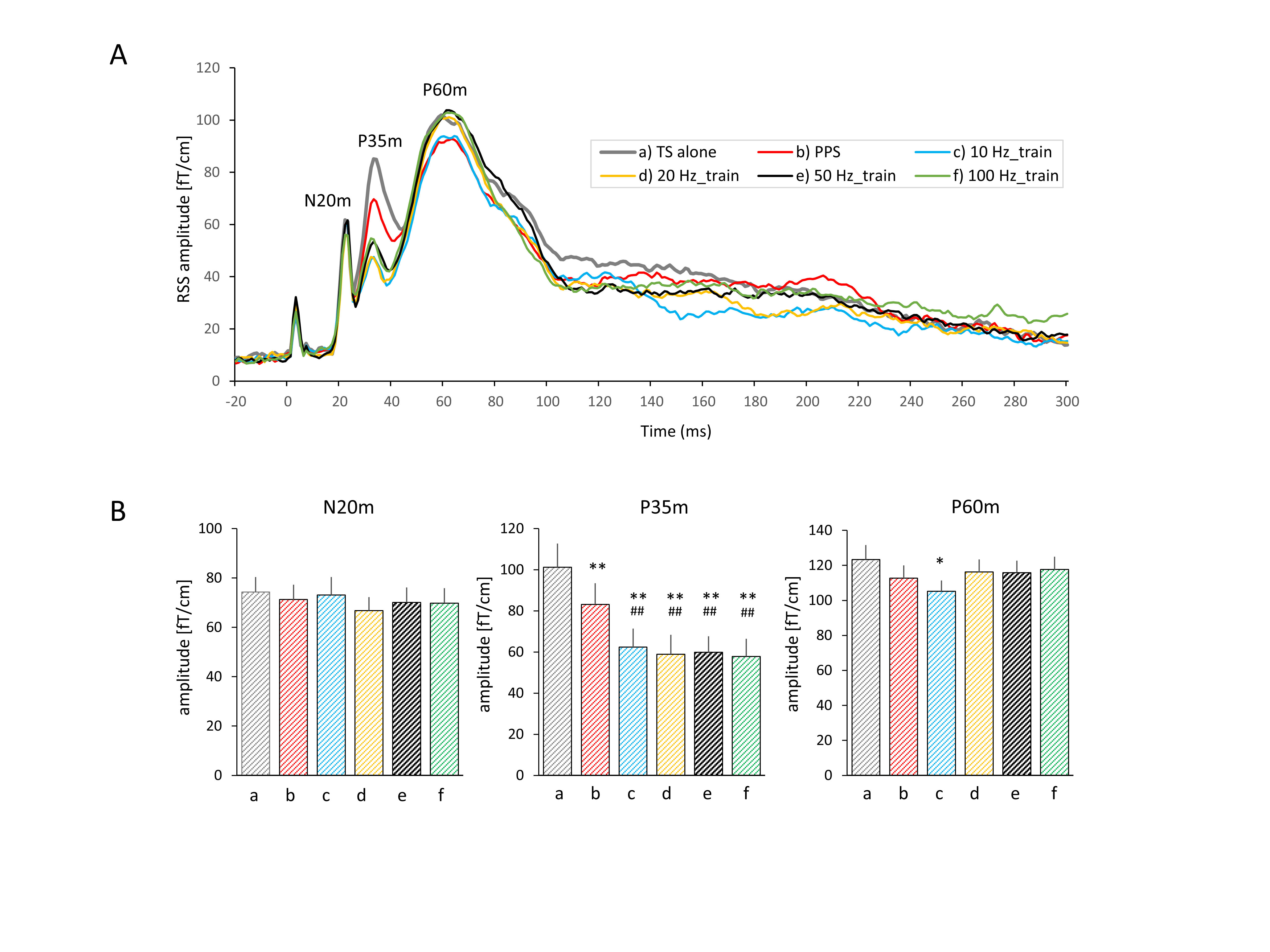

Supplement: Supplementary file 1 — Supplementary Figure 1 Grand averaged RSS waveforms and the mean amplitudes of prominent deflections. (A) Time-courses of the grand averaged RSS waveforms from 20 ms before to 300 ms after the test stimulation elicited by all conditions are superimposed. The gray, red, blue, orange, black, and green lines indicate the RSS waveforms elicited by condition_a, _b, _c, _d, _e, and _f respectively. (B) The mean RSS amplitude at N20m, P35m, and P60m. The a, b, c, d, e, and f under each bar graph indicate condition_a, _b, _c, _d, _e, and _f respectively. The error bars indicate the standard error of the mean (SEM). *p < 0.05 (vs. condition_a), **p < 0.01 (vs. condition_a), ##p < 0.01 (vs. condition_b). (TIF 1811 KB) [file 10548_2018_648_MOESM1_ESM.tif]

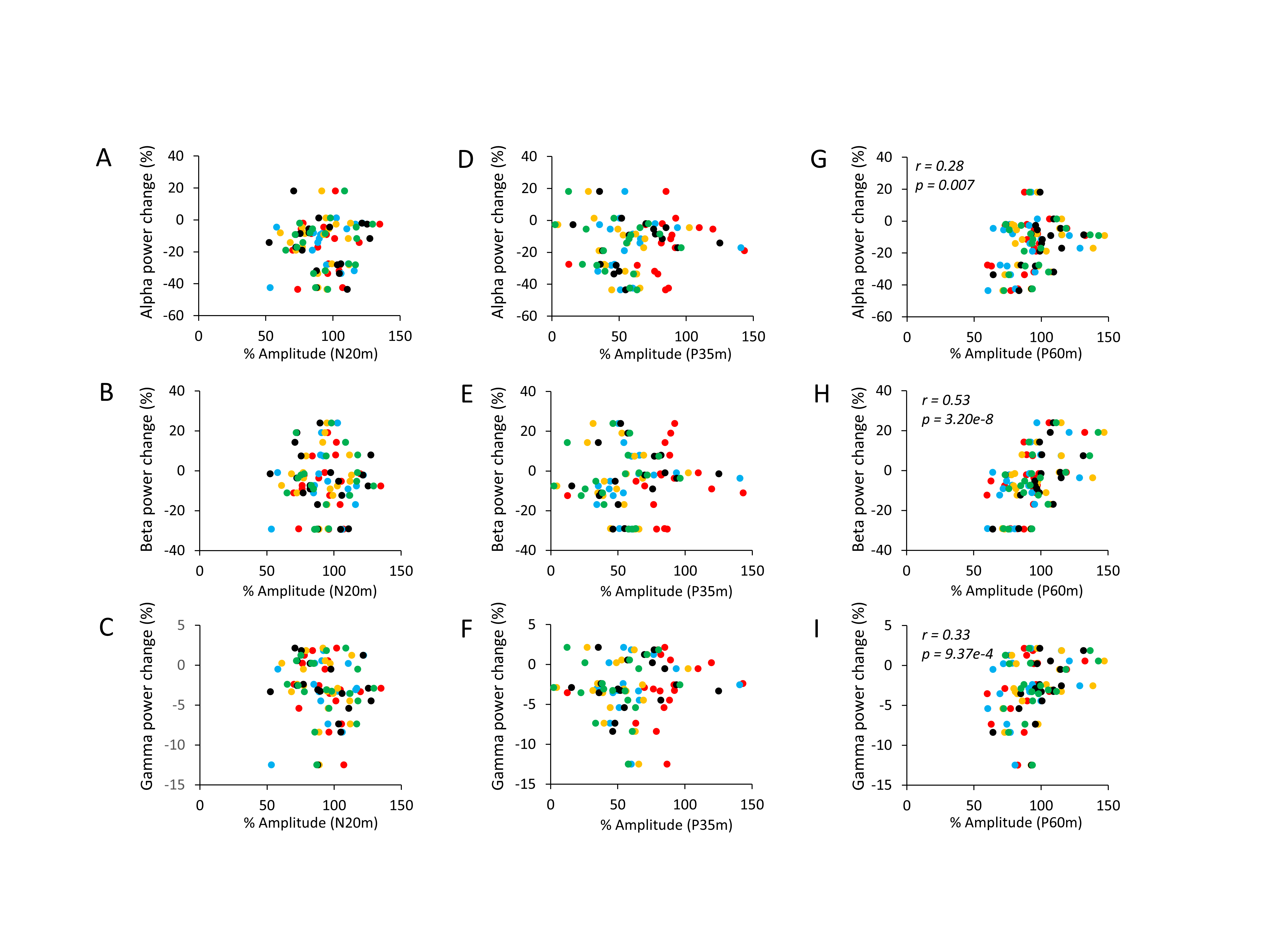

Supplement: Supplementary file 2 — Supplementary Figure 2 Pearson’s product-moment correlation coefficients (r) between the PPD ratio at N20m, P35m, and P60m induced by all conditions with conditioning stimulation (condition_b, _c, _d, _e, and _f), and changes in power for each frequency band (alpha, beta, and gamma) induced by test stimulation under condition_a. (A) The relationship between alpha power changes and the PPD ratio at N20m, (B) beta power changes, and the PPD ratio at N20m, (C) gamma power changes and the PPD ratio at N20m, (D) alpha power changes and the PPD ratio at P35m, (E) beta power changes and the PPD ratio at P35m, (F) gamma power changes and the PPD ratio at P35m, (G) alpha power changes and the PPD ratio at P60m, (H) beta power changes and the PPD ratio at P60m, and (I) gamma power changes and the PPD ratio at P60m. Statistically significant positive correlations were observed between the PPD ratio at P60m and alpha, beta, and gamma power changes, whereas no significant correlation was observed at N20m or P35m. The red, blue, orange, black, and green dots indicate the data elicited by conditions_b, _c, _d, _e, and _f respectively. (TIF 1396 KB) [file 10548_2018_648_MOESM2_ESM.tif]

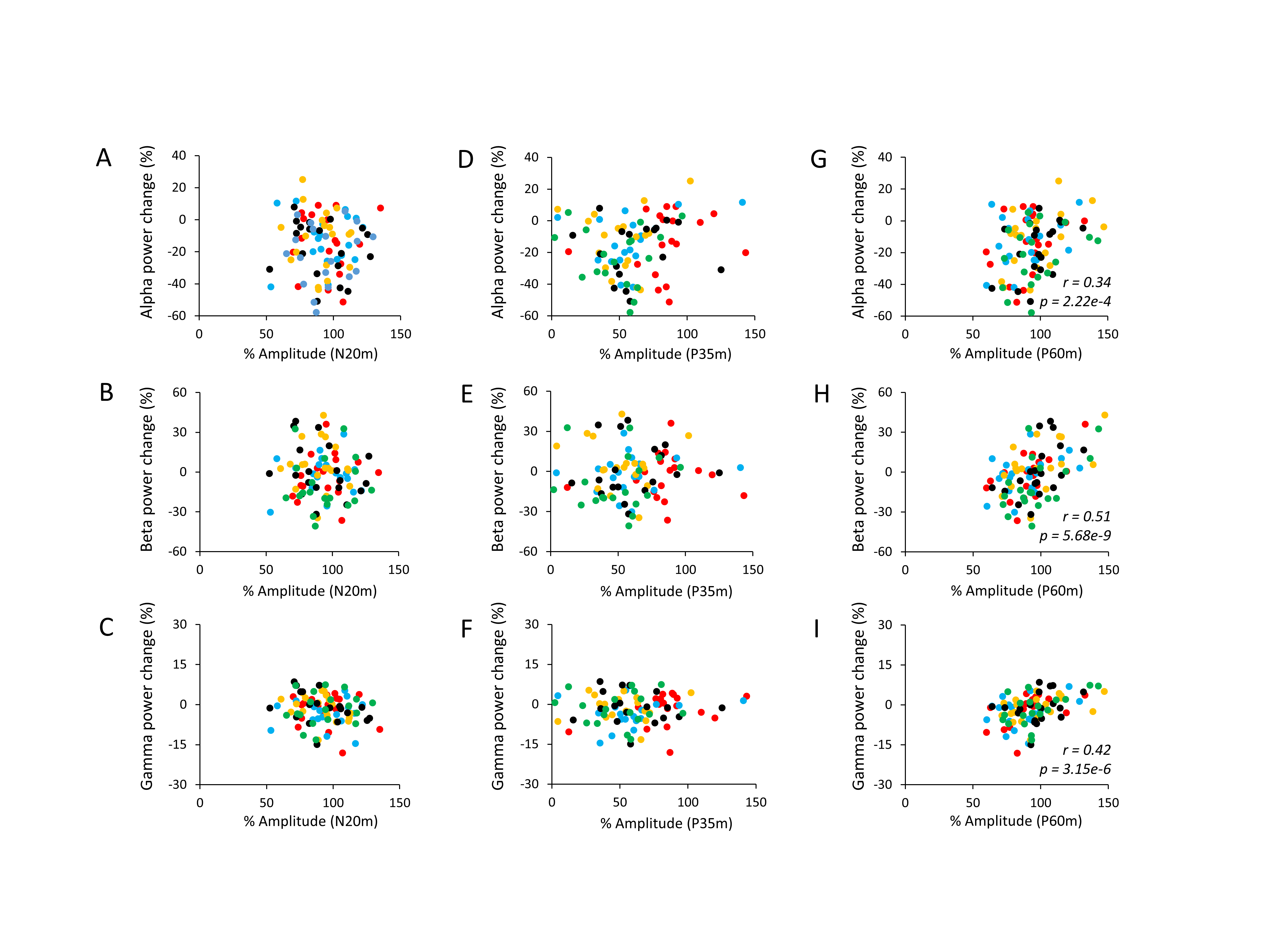

Supplement: Supplementary file 3 — Supplementary Figure 3 Pearson’s product-moment correlation coefficients (r) between the PPD ratio at N20m, P35m, and P60m induced under all conditions with conditioning stimulation (condition_b, _c, _d, _e, and _f), and changes in power for each frequency band immediately before test stimulation, following each conditioning stimulation (condition_b, _c, _d, _e, and _f). A) N20m vs. alpha, B) N20m vs. beta, C) N20m vs. gamma, D) P35m vs. alpha, E) P35m vs. beta, F) P35m vs. gamma, G) P60m vs. alpha, H) P60m vs. beta, and I) P60m vs. gamma. Positive correlations were observed between the PPD ratio at P60m and alpha, beta, and gamma power changes, while no significant correlation was observed at N20m or P35m. The red, blue, orange, black, and green dots indicate the data elicited under condition_b, _c, _d, _e, and _f respectively. (TIF 1413 KB) [file 10548_2018_648_MOESM3_ESM.tif]

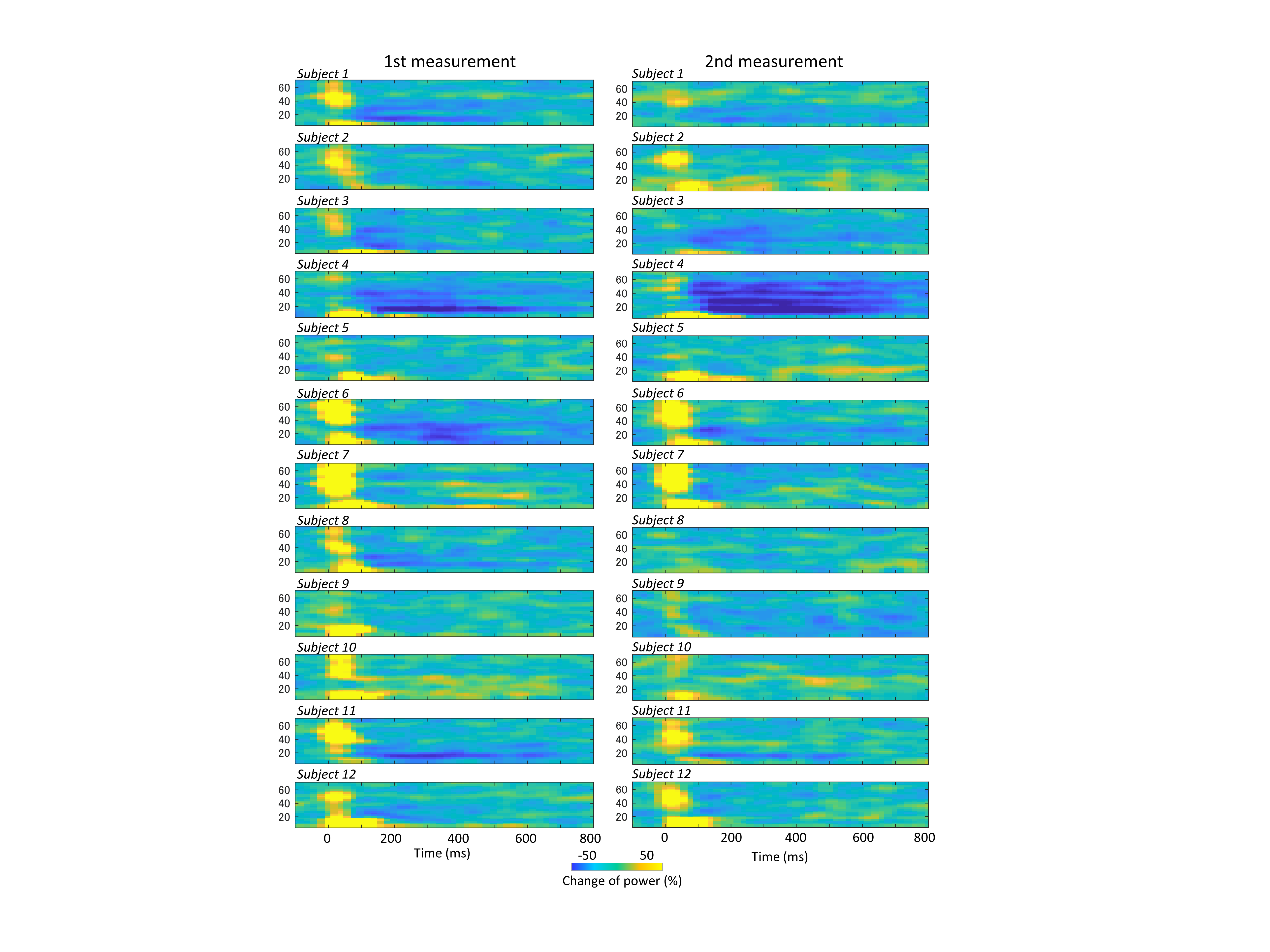

Supplement: Supplementary file 4 — Supplementary Figure 4 Time frequency maps of signal power changes induced by condition_a (TS_alone) from 100 ms before to 800 ms after the test stimulation in all subjects. The left column shows the maps induced by the first measurement, and the right column shows the maps induced by the second measurement in the same subject. (TIF 1982 KB) [file 10548_2018_648_MOESM4_ESM.tif]

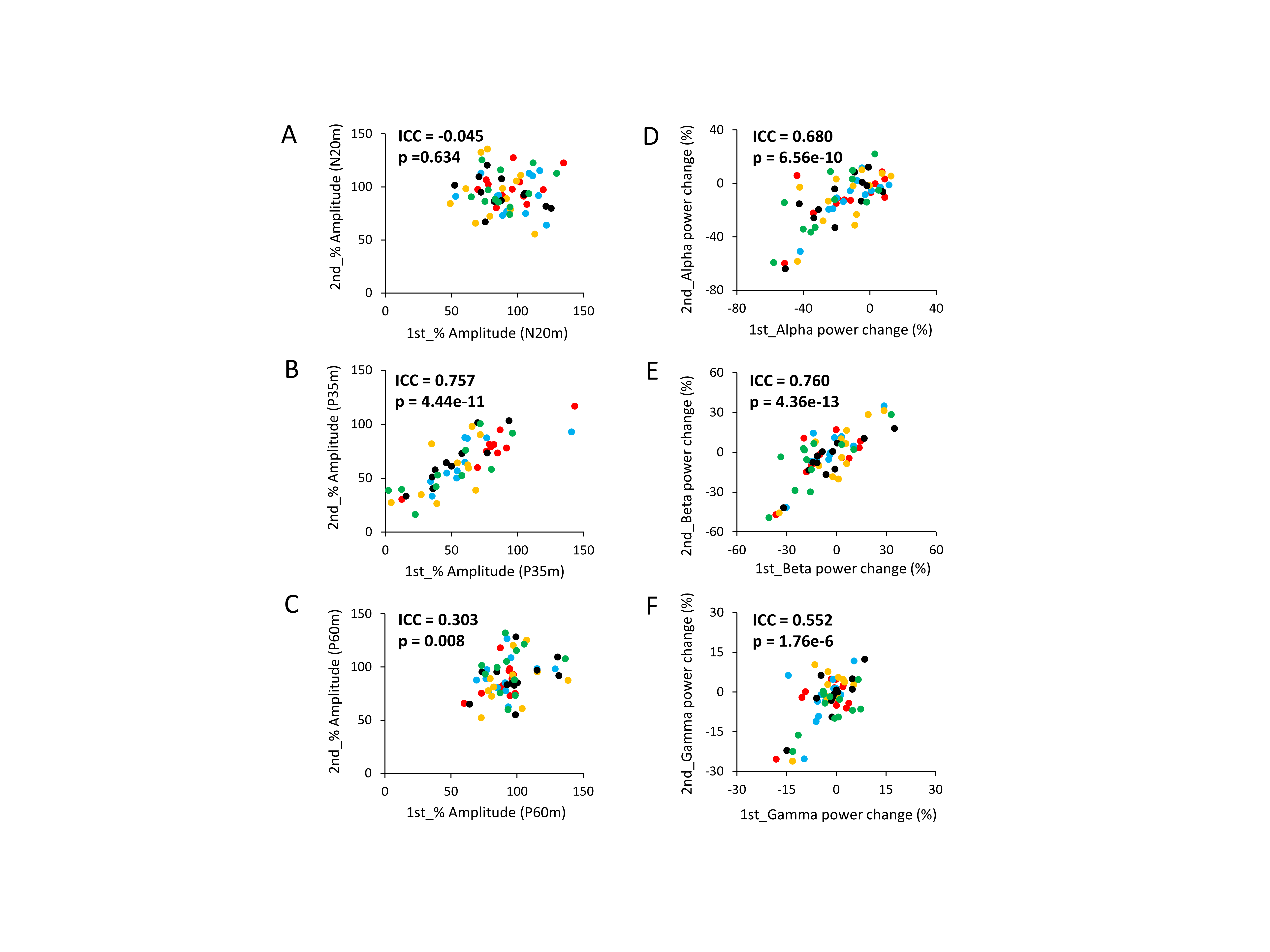

Supplement: Supplementary file 5 — Supplementary Figure 5 Pearson’s product-moment correlation coefficients (r) between the test and retest PPD ratio at N20m, P35m, and P60m and the alpha, beta, and gamma power changes immediately before test stimulation, following each conditioning stimulation (condition_b, _c, _d, _e, and _f). A) N20m, B) P35m, C) P60m, D) alpha power changes, E) beta power changes, and F) gamma power changes. The red, blue, orange, black, and green dots indicate the data obtained for condition_b, _c, _d, _e, and _f respectively. Significant positive correlation in the PPD ratio between the test and retest measurements was noted; ICC values were excellent for P35m, but poor for P60m. Significant positive correlations in the alpha, beta, and gamma power changes between test and retest measurements were noted; the ICC values were good for alpha, excellent for beta, and fair for gamma power changes. (TIF 1198 KB) [file 10548_2018_648_MOESM5_ESM.tif]
